# Long non-coding RNA CARLo-5 promotes tumor progression in hepatocellular carcinoma via suppressing miR-200b expression

**DOI:** 10.18632/oncotarget.19597

**Published:** 2017-07-26

**Authors:** Chunqing Dou, Liyuan Sun, Xin Jin, Mingming Han, Bao Zhang, Xian Jiang, Jinyong Lv, Tao Li

**Affiliations:** ^1^ Department of Hepatobiliary Surgery, The First Affiliated Hospital of Chinese PLA General Hospital, Beijing, China

**Keywords:** hepatocellular carcinoma, CARLo-5, EZH2, miR-200b, epithelial-mesenchymal transition

## Abstract

Long non-coding RNAs (lncRNAs) play key roles in cancer initiation and progression. The aim was to investigate the biological functions and clinical significance of long non-coding RNA CARLo-5 in hepatocellular carcinoma (HCC). QRT-PCR was performed to investigate CARLo-5 expression in HCC tissues and cells. Kaplan-Meier curve and multivariate analysis validated the association between CARLo-5 expression and overall survival (OS) in HCC patients. Cell proliferation and invasion was performed by CCK8 cell proliferation, cell colony formation and transwell invasion assays. Western-blot assay was performed to evaluate the protein expression of Twist1, ZEB1, E-cadherin and Vimentin. Tumor xenografts were performed to evaluate the effect of CARLo-5 on tumor growth *in vivo*. RNA Immunoprecipitation (RIP) and Chromatin Immunoprecipitation (ChIP) were also performed. Our results showed that CARLo-5 expression was significantly higher in HCC tissues and upregulated CARLo-5 expression was closely correlated with tumor size and advanced tumor stage. Kaplan-Meier curve and multivariate analysis validated that higher CARLo-5 expression predicted a poor prognosis for HCC patients and was an independent risk factor for OS in HCC patients. *In vitro*, knockdown of CARLo-5 inhibited cell proliferation, colony formation, cell invasion and inhibited the cell epithelial-mesenchymal transition (EMT) by up-regulating the E-cadherin expression and down-regulating Twist1, ZEB1 and vimentin expression in HCC cells. Furthermore, we demonstrated that CARLo-5 inhibited the miR-200b expression via EZH2. *In vivo*, knockdown of CARLo-5 significantly inhibited the tumor growth. Thus, our results indicated that CARLo-5 represented a novel tumor biomarker and therapeutic target for HCC.

## INTRODUCTION

Hepatocellular carcinoma (HCC) is the most commonly diagnosed malignancy in the liver and ranks the third most frequent cancer-related mortality in the world [1, 2]. About 80% of HCC patients are diagnosed at an advanced stage of disease, due to the lack of symptoms during early stages of HCC and the rapid progression of disease. Because the high rates of recurrence and metastasis, the five-year survival rate of less than 5% in HCC patients that was diagnosed with unresectable disease [3]. Thus, the effective methods for the HCC patients’ treatment are needed.

Long noncoding RNAs (LncRNAs) are a recently discovered class of endogenous RNA that more than 200 nucleotides in length, which play important roles in carcinogenesis and progression [4]. LncRNAs functions through a variety of mechanisms, such as, recruiting regulating gene expression, scaffolding that assemble chromatin remodeling machinery at the site of regulation, mediators of alternative splicing, precursors for small ncRNAs, and so on [5]. Abnormal expression of lncRNAs have been found to be associated with hepatocarcinogenesis and play key roles in progression and metastasis [6]. Highly up-regulated in liver cancer (HULC) was originally identified as the most overexpressed long non-coding RNA in hepatocellular carcinoma. Up-regulated HULC by HBx promoted proliferation of hepatoma cells through regulating a tumor suppressor gene p18 located near HULC in the same chromosome [7]. Studies showed that lncRNA-hPVT1 was up-regulated in HCC tissues and patients with higher lncRNA-hPVT1 expression had a poor clinical prognosis. In addition, the transforming growth factor (TGF)-β1/lncRNA-hPVT1/NOP2 pathway was promised in the progression of HCC [8]. Patients with HOTAIR expression had significantly poorer over survival time and a larger primary tumor size than those without HOTAIR expression [9]. These above results indicated LncRNAs play potential roles in HCC.

Cancer-associated region long noncoding RNAs (CARLos) was located in the 8q24 region. CARLo-5, one of the long noncoding RNAs, was significantly correlated with the rs6983267 allele associated with increased cancer susceptibility and has a function in cell-cycle regulation and tumor development [10]. Long non-coding RNA CARLo-5 was also found to act as a negative prognostic factor and exhibits tumor pro-oncogenic activity in non-small cell lung cancer [11]. Zhang *etal* found knockdown of CARLo-5 in gastric cancer cell lines significantly inhibited the cell proliferation via inducing G0/G1 cell-cycle arrest and apoptosis. Furthermore, the ERK/MAPK pathway was found to be inactivated in the gastric cells after CARLo-5 knockdown [12]. However, the biological functions and clinical significance of long non-coding RNA CARLo-5 in hepatocellular carcinoma (HCC) still to be investigated.

In the study, we showed that CARLo-5 expression levels were significantly higher in HCC patient samples and higher levels of CARLo-5 predicted poor prognosis for HCC patients. Functional studies revealed that knockdown of CARLo-5 could inhibit cell proliferation, invasion and epithelial-mesenchymal transition (EMT). Mechanism research demonstrated that CARLo-5 interacted with EZH2 and regulated the miR-200b expression. In addition, we demonstrated that CARLo-5 inhibited the tumor growth *in vivo*. Therefore, our present study indicated that CARLo-5 may represent a novel tumor marker and therapeutic targets for HCC.

## RESULTS

### The expressions of CARLo-5 in human HCC tissue samples and cells

**Figure 1:**
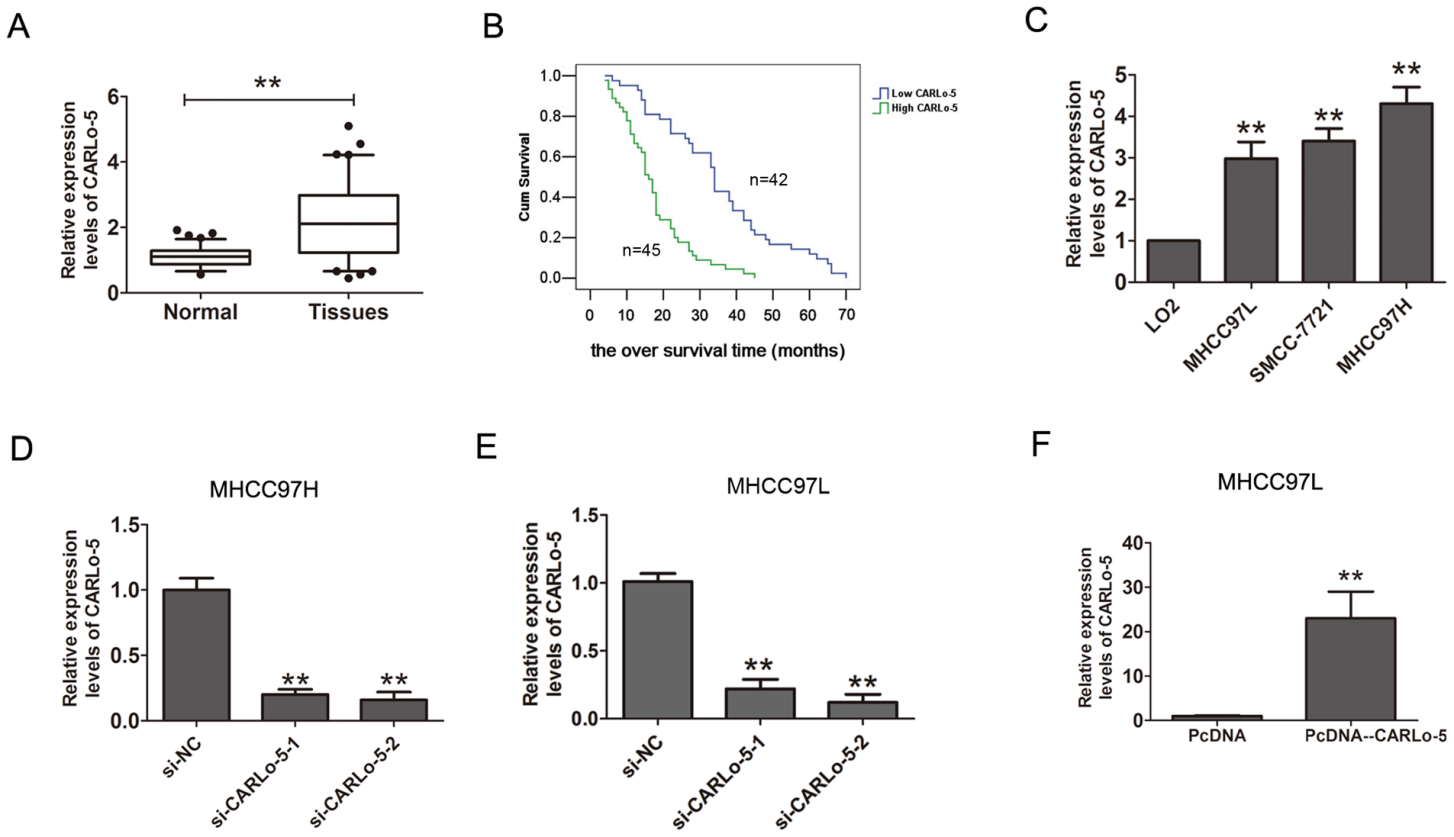
The expression levels of CARLo-5 in human HCC tissue samples and cells **(A)** CARLo-5 was detected by qRT-PCR assays in HCC tissues and adjacent normal tissues. The CARLo-5 was normalized to GADPH. Paired samples t-test was performed to analyze the differences between two group samples. **(B)** Kaplan-Meier curve analysis and log-rank test was performed to analyze the association between the expressions of CARLo-5 and the over survival time in HCC patients. **(C)** Relative expression of CARLo-5 was detected by qRT-PCR assays in HCC cells (MHCC97H, MHCC97L and SMCC-7721) and a normal liver cell LO2. The expression of CARLo-5 was normalized to GADPH. **(D-E)** Relative expression of CARLo-5 was detected by qRT-PCR assays after knockdown of CARLo-5 by si- CARLo-5-1 or si- CARLo-5-2 in MHCC97H and MHCC97L cells. **(F)** Relative expression of CARLo-5was detected by overexpression of CARLo-5 by pcDNA-CARLo-5 plasmid in MHCC97L. Error bars represent the mean ± S.D. of triplicate experiments. **P<0.05.

The expression levels of CARLo-5 in human HCC tissues and cell lines were analyzed by qRT-PCR methods. As shown in Figure 1A, the results showed that CARLo-5 expression levels were significantly up-regulated in HCC tissues compared with the adjacent normal tissues. Furthermore, according the median expression of CARLo-5 in HCC tissues (2.45), the CARLo-5 expression levels were classified two groups (higher expression group and lower expression group), the results showed that CARLo-5 expression was positively correlated with the larger tumor size and AJCC stage, but no correlation with age, gender, and so on (P<0.05, Table 1). Besides, as shown in Table 2, univariate and multivariate analysis revealed that larger tumor size, AJCC stage and CARLo-5 expression were independent prognostic factors for the overall survival (OS) time in HCC patients. The Kaplan-Meier analysis and log-rank test were used to evaluate the relationship between CARLo-5 expression and over survival time in HCC patients. The results demonstrated that higher expression of CARLo-5 in HCC patients predicted a poor prognosis for HCC patients (Figure 1B). In addition, we also detected the expression CARLo-5 in HCC cell line and the results showed that the CARLo-5 expression levels were dramatically increased in three HCC cells than that in the normal liver cell line LO2 (Figure 1C). Collectively, these results suggested that up-regulation of CARLo-5 could be involved in development, progression and prognosis of HCC.

**Table 1:** Correlation between the lncRNA CARLo-5 expression and clinicopathological feathers of HCC patients

Characteristics	Patients number(n=87)	The CARLo-5 expression		p Value
		Low (n=42)	High (n=45)	
**Gender**				0.945
Male	66	32	34	
Female	21	10	11	
**Age**				0.496
≤60	59	27	32	
>60	28	15	13	
**Tumor size**				0.001**
<5cm	44	29	15	
>5cm	43	13	30	
**Histological grade**				0.824
Well	37	18	19	
moderately	29	15	14	
poor	21	9	12	
**Vein invasion**				0.561
No	49	25	24	
Yes	38	17	21	
**AFP(ng/ml)**				0.172
<400	29	17	12	
>400	58	25	33	
**HBV infection**				0.080
Yes	69	30	39	
No	18	12	6	
**AJCC stage**				0.004**
I-II	46	29	17	
III-IV	41	13	28	

**, P < 0.05.

**Table 2:** Univariate and multivariate analysis of the over survival (OS) time in 87 cases HCC patients

Variables	Univariate analysis			Multivariate analysis		
	HR	95%CI	p Value	HR	95%CI	p Value
Gender	1.131	0.707-1.777	0.218			
Age	1.044	0.976-1.145	0.502			
Tumor size(cm)	2.445	1.228-3.873	0.001**	2.012	1.352-3.458	0.001**
Histological grade	0.986	0.455-1.977	0.641			
Vein invasion	1.617	0.973-2.664	0.065			
AFP(ng/ml)	1.138	0.709-1.539	0.455			
HBV infection	1.332	0.992-1.879	0.216			
AJCC stage	1.917	1.232-2.996	0.003**	1.754	1.146-2.729	0.011**
CARLo-5	3.024	1.839-4.368	0.001**	2.665	1.457-3.679	0.001**

HR: hazard ratio, CI: confidence interval, **, p<0.05.

### Knockdown of CARLo-5 inhibits the cell proliferation and invasion in HCC

**Figure 2:**
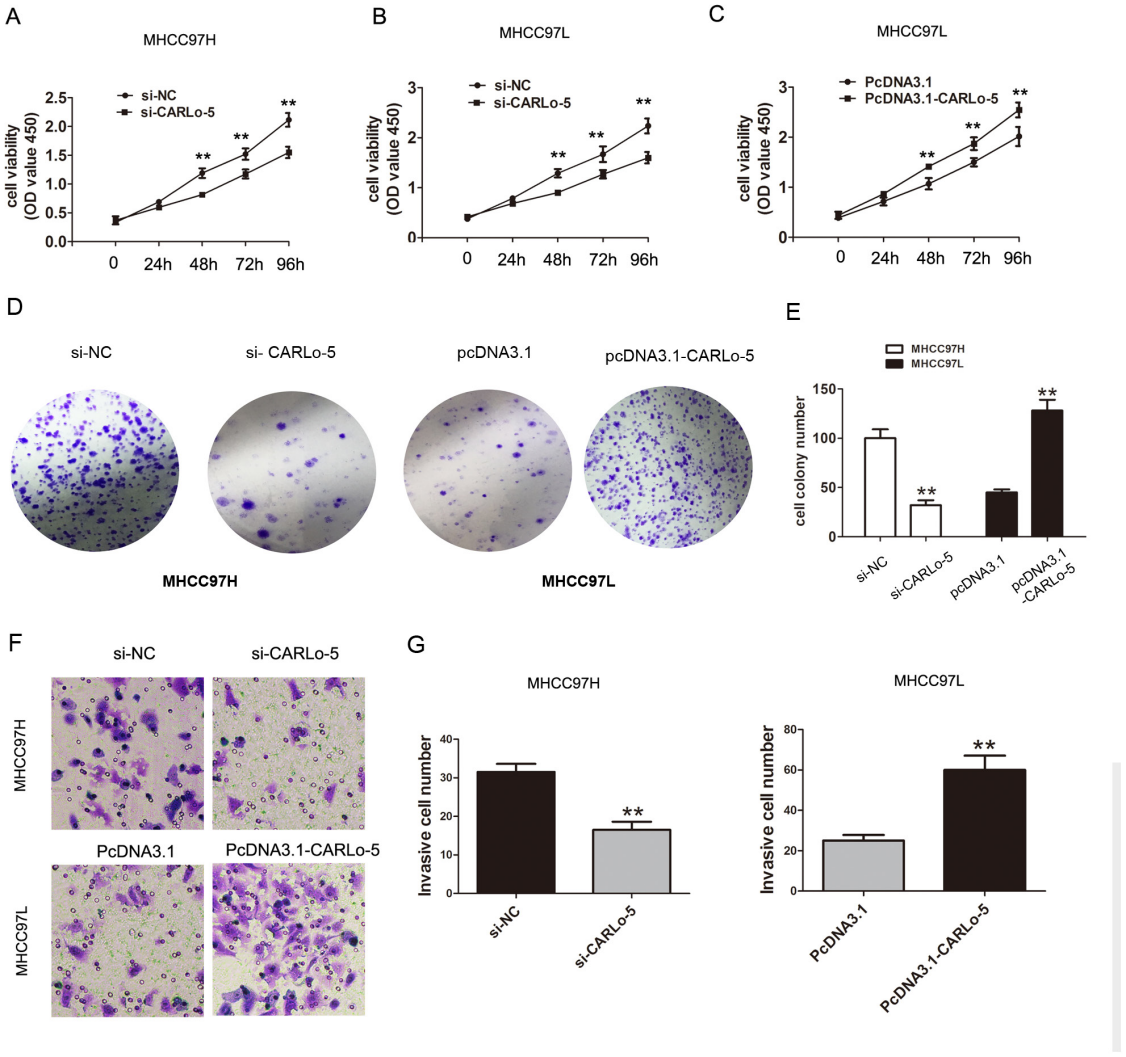
The association of CARLo-5 and cell proliferation and cell invasion in HCC **(A-C)** The cell proliferation was showed by CCK8 assays after knockdown of CARLo-5 by si-CARLo-5 in MHCC97H and MHCC97L cells or overexpression of CARLo-5 by pcDNA-CARLo-5 in MHCC97L. **(D-E)** The cell colony and cell number was detected by cell colony formation assays after knockdown of CARLo-5 by si-CARLo-5 in MHCC97H or overexpression of CARLo-5 by pcDNA-CARLo-5 in MHCC97L. **(F-G)** The cell invasion ability was showed by transwell assays and cell invasion number analysis after knockdown of CARLo-5 by si-CARLo-5 in MHCC97H cells or overexpression of CARLo-5 by pcDNA-CARLo-5 in MHCC97L. Error bars represent the mean ± S.D. of triplicate experiments. **P<0.05.

To explore the potential biological significance of CARLo-5 in HCC, we evaluated the effects of CARLo-5 on the cell proliferation colony formation and invasion by performing loss-and gain-off function investigations. The knockdown efficiency of siRNA-CARLo-5-1 and siRNA-CARLo-5-2 in MHCC97H and MHCC97L cell lines was shown in Figure 1D and 1E. The siRNA-CARLo-5-2 was introduced into HCC cell lines in the subsequent experiments due to higher knockdown efficiency for CARLo-5. The pcDNA3.1-CARLo-5 was transfected into MHCC97L cells to increase the CARLo-5 expression levels (Figure 1F). Our results showed that CARLo-5-1 knockdown inhibited the cell proliferation in MHCC97H cells using CCK8 cell proliferation assays, but cell proliferation abilities was enhanced by over-expression of CARLo-5 in MHCC97L cells (Figure 2A-2C). CARLo-5-1 knockdown also inhibited the cell colonies number in MHCC97H cells, but cell colonies number was enhanced by over-expression of CARLo-5 in MHCC97L cells (Figure 2D-2E). The transwell cell invasion assay was used to evaluate to cell invasion ability and the results showed that the cell invasive number was significantly reduced after CARLo-5 knockdown, but was increased by introducing pcDNA3.1- CARLo-5 into MHCC97L (Figure 2F-2G). Thus, these results suggested that CARLo-5 exerted tumor-promoted effects in human HCC cells and inhibited the tumor progression.

### Knockdown of CARLo-5 inhibits the cells epithelial - mesenchymal transition

**Figure 3:**
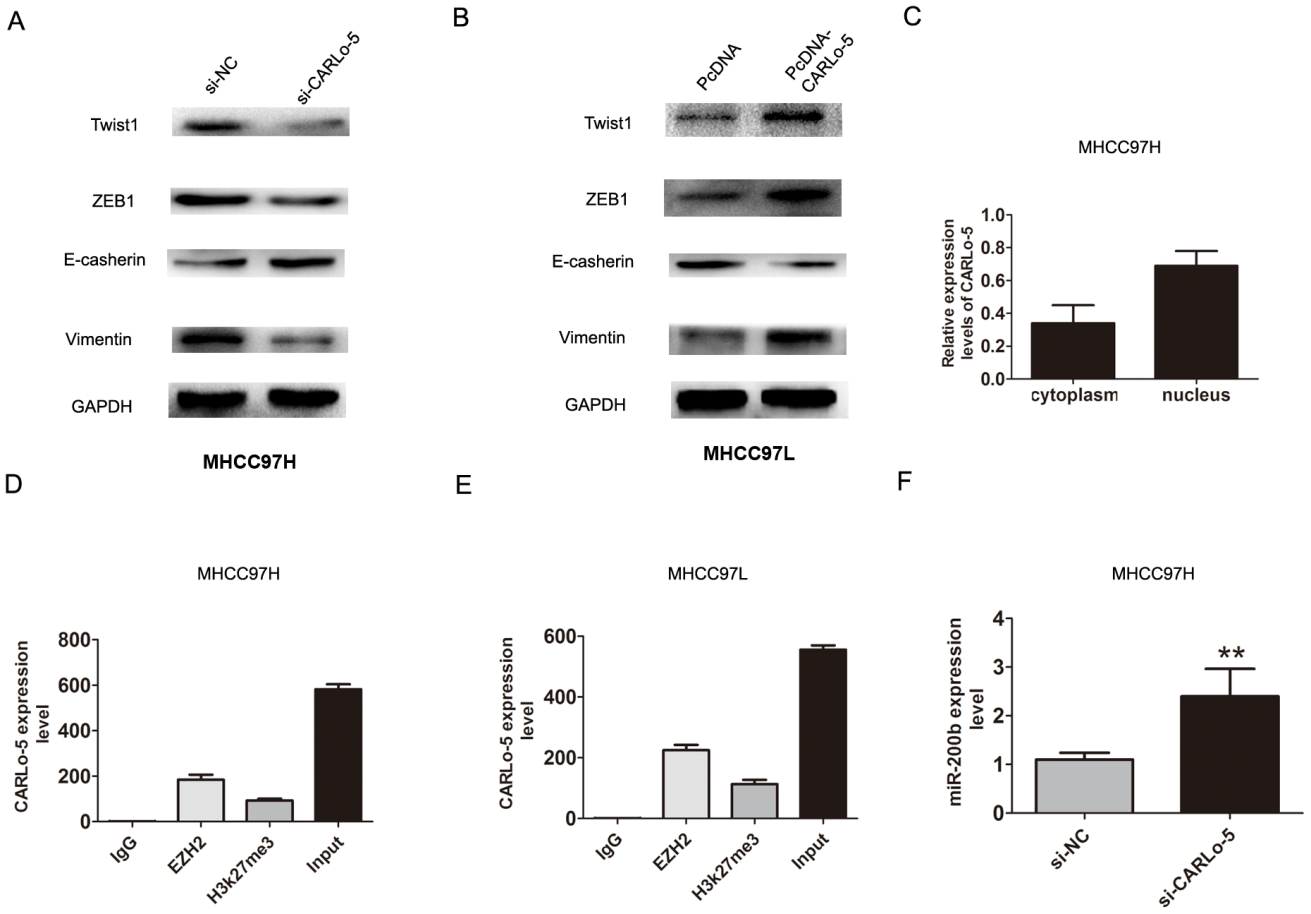
The association of CARLo-5 and cell EMT in HCC cells **(A-B)** The protein expression of Twist1, ZEB1, E-cadherin and Vimentin were showed by western blot assays after knockdown of CARLo-5 by si-CARLo-5 in MHCC97H cells or overexpression of CARLo-5 by pcDNA-CARLo-5 in MHCC97L. **(C)** The expression level of CARLo-5 was detected in the cytoplasm and nucleus inMHCC97H cells. **(D-E)** RIP assays was used to detect the CARLo-5 with anti- IgG, anti-EZH2, anti-H3k27me3 and input were presented in MHCC97H or MHCC97L cells. **(F)** The mRNA expression of miR-200b was showed by qRT-PCR assays after knockdown of CARLo-5 by si-CARLo-5 in MHCC97H cells. The CARLo-5 was normalized to U6. Error bars represent the mean ± S.D. of triplicate experiments. **P<0.05.

Epithelial-mesenchymal transition (EMT) process is the remarkable presentation for tumor cell invasion and metastasis [13]. We explored that whether CARLo-5 expression was associated with HCC cell EMT process. The western blot analysis confirmed that Twist1, ZEB1, Vimentin were down-regulated after CARLo-5 knockdown while E-cadherin was up-regulated in MHCC97H cells (Figure 3A). However, over-expression of CARLo-5 in MHCC97L cells significantly increased the expression levels of Twist1, ZEB1, Vimentin, but inhibiting the E-cadherin in the MHCC97L cells (Figure 3B). Therefore, these results indicated that inhibition of CARLo-5 led to the reversal of EMT to MET phenotype.

### Overexpression of CARLo-5 inhibits miR-200b expression by interacted with EZH2

Previous studies had shown that lncRNAs involved in regulation of cancer cells phenotype through activation of oncogenes or inactivation of tumor suppressors by binding with specific RNA-binding proteins [14]. For example, MALAT1 or UCA1 could play important roles in facilitating genome-wide occupancy of EZH2 onto chromatin in tumor cells [15, 16]. To detect the function of CARLo-5, we detected the distribution of CARLo-5 expression in MHCC97H cells. The results demonstrated that CARLo-5 was enriched in both nucleus and cytoplasm, according to the GAPDH and U1, the nucleus/cytoplasm separation (Figure 3C). Next, we detected whether EZH2 specifically binds to CARLo-5 in HCC cells by performing RIP-qPCR analysis in MHCC97H and MHCC97L cells using EZH2 and H3k27me3 antibodies. The data verified that CARLo-5 bound to EZH2 and H3k27me3 in MHCC97H and MHCC97L cells (Figure 3D-3E).

**Figure 4:**
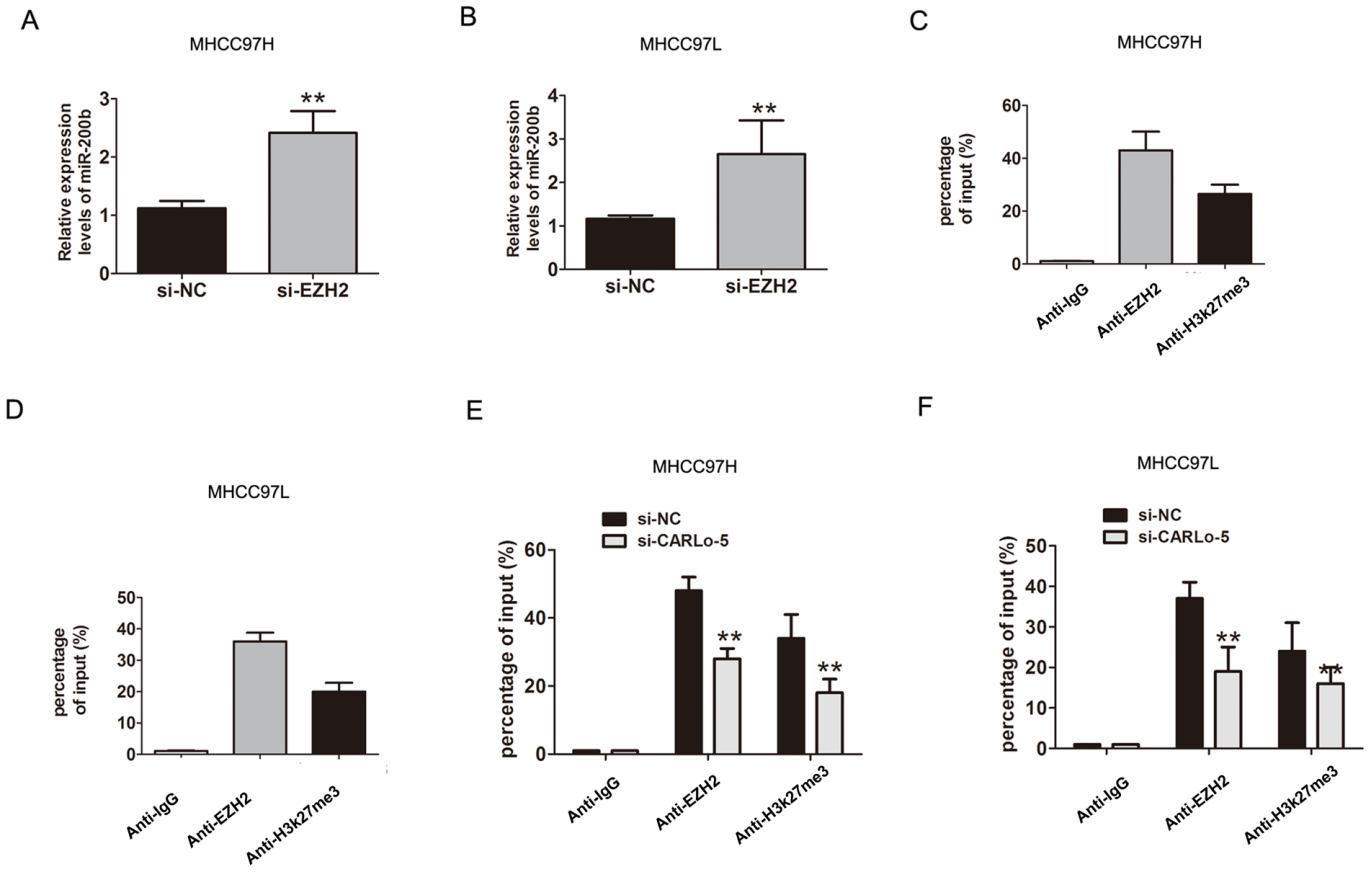
CARLo-5 inhibited the miR-200b expression by EZH2 in HCC **(A-B)** The mRNA expression of miR-200b was showed by qRT-PCR assays after knockdown of EZH2 by si-EZH2 in MHCC97H cells or MHCC97L cells. **(C-D)** CHIP assays was used to detect the miR-200b expression with anti- IgG, anti-EZH2, anti-H3k27me3 antibodies in MHCC97H or MHCC97L cells. **(E-F)** CHIP assays was used to detect the miR-200b expression with anti- IgG, anti-EZH2, anti-H3k27me3 antibodies after knockdown of CARLo-5 in MHCC97H cells or MHCC97L cells. Error bars represent the mean ± S.D. of triplicate experiments. **P<0.05.

EZH2 is a core subunit of PRC2 that promoted gene silencing via its histone methyltransferase activity [17]. To investigate the role of CARLo-5 on EZH2-mediated Polycomb-dependent gene silencing, we found that miR-200b, as a target with tumor suppressor function of EZH2 [18]. The results showed that the miR-200b was up-regulated after CARLo-5 knockdown in MHCC97H (Figure 3F). Furthermore, after knockdown of EZH2, the results also showed that the expression of miR-200b was significantly increased in MHCC97H and MHCC97L cells (Figure 4A-4B). Thus, based on the above these results, we speculated that miR-200b may be involved in the contributions of CARLo-5 to HCC progression. To demonstrate the involvement of PRC2 at the level of the genome and chromatin modifications, the ChIP assays was performed using antibodies for EZH2 and H3K27me3. The results demonstrated that EZH2 could directly bind to miR-200b promoter region and mediate H3K27me3 demethylation modification in MHCC97H and MHCC97L cells (Figure 4C-4D). However, knockdown of CARLo-5 resulted in reducing their binding ability and EZH2 and H3K27me3 demethylation modification (Figure 4E-4F). These data indicated that CARLo-5 contributed to HCC cell progression partly through repressing miR-200b expression.

### MiR-200b partly mediates the function of CARLo-5 in HCC cell invasion capacity

**Figure 5:**
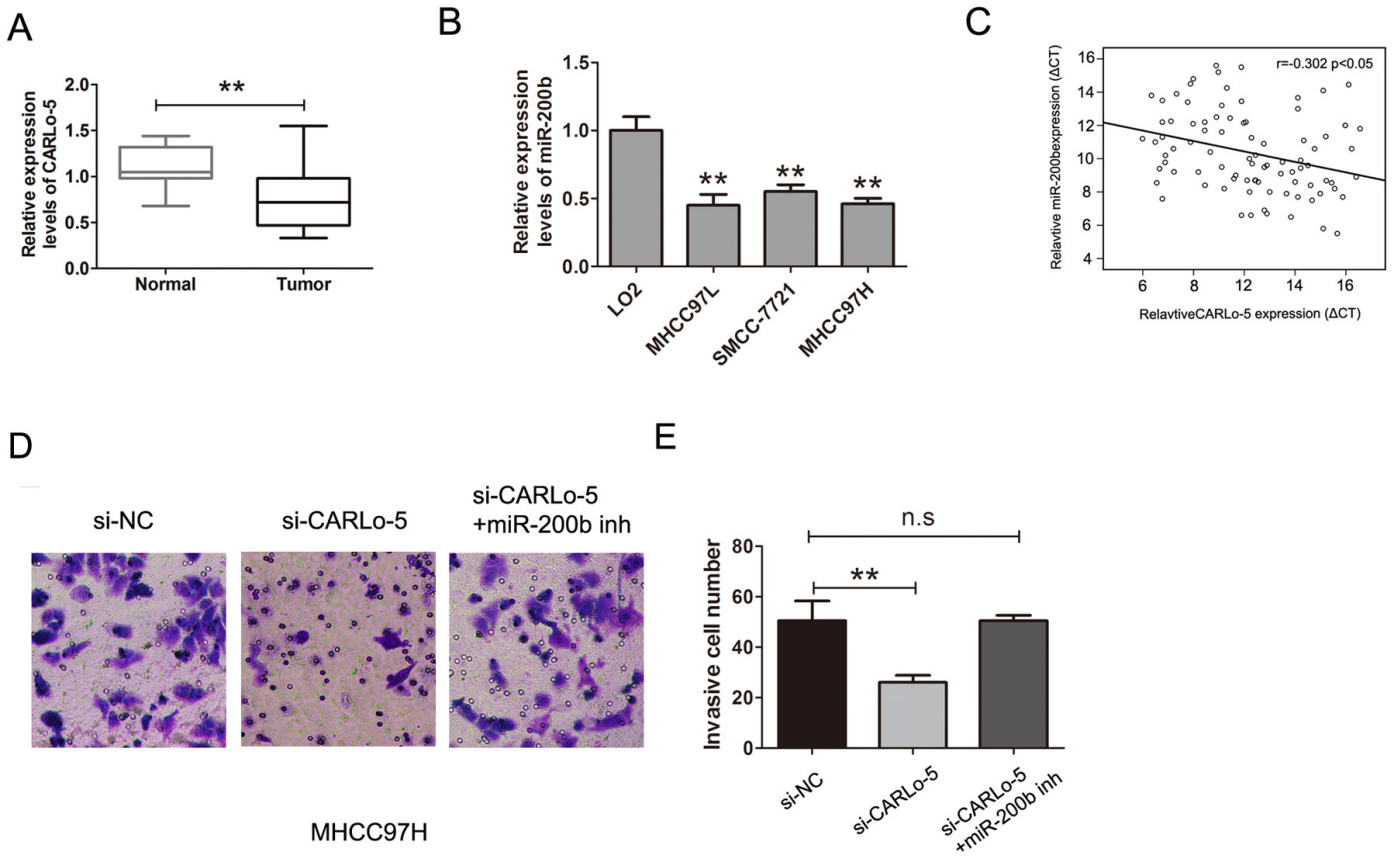
CARLo-5 promoted the cell invasion by miR-200b in HCC **(A)** MiR-200b was detected by qRT-PCR assays in HCC tissues and adjacent normal tissues. The miR-200b was normalized to U6. **(B)** MiR-200b was detected by qRT-PCR assays in HCC cells and LO2 cells. The miR-200b was normalized to U6. **(C)** The association between the expression of miR-200b and CARLo-5 in HCC tissues was analyzed by Pearson correlation coefficient. **(D-E)** The cell invasion abilities was showed by transfecting si-NC, si- CARLo-5 or si- CARLo-5+miR-200b inhibitor. Error bars represent the mean ± S.D. of triplicate experiments. **P<0.05.

Members of the miR-200 family (miR-200a and miR-200b) play important roles in HCC cell migration and invasion by regulating E-cadherin expression and suppressed hepatocellular carcinoma metastasis [19, 20]. QRT-PCR analysis was used to determine expression levels of miR-200b in HCC tissues and adjacent normal tissues, we found that miR-200b was down-regulated in HCC tissues and the levels of miR-200b expression were also significantly lower in HCC cells than that observed in normal liver cell LO2 (Figure 5A-5B). The expression of CARLo-5 in HCC tissues was negatively associated with miR-200b expression (Figure 5C, P<0.05, r=-0.302). To determine whether miR-200b was involved in CARLo-5 induced promotion of HCC cell invasion, we transfected the si-CARLo-5 into MHCC97H cell, the transwell cell invasion assays and analysis also demonstrated that cell invasion abilities was suppressed by knockdown of CARLo-5, whereas, the effects were reversed by co-transfection of si-CARLo-5 and miR-200b inhibitor in MHCC97H (Figure 5D-5E).Therefore, these results revealed that CARLo-5 regulated HCC cell invasion partly through the down-regulation of miR-200b expression.

### Knockdown of CARLo-5 inhibits tumor growth *in vivo*

**Figure 6:**
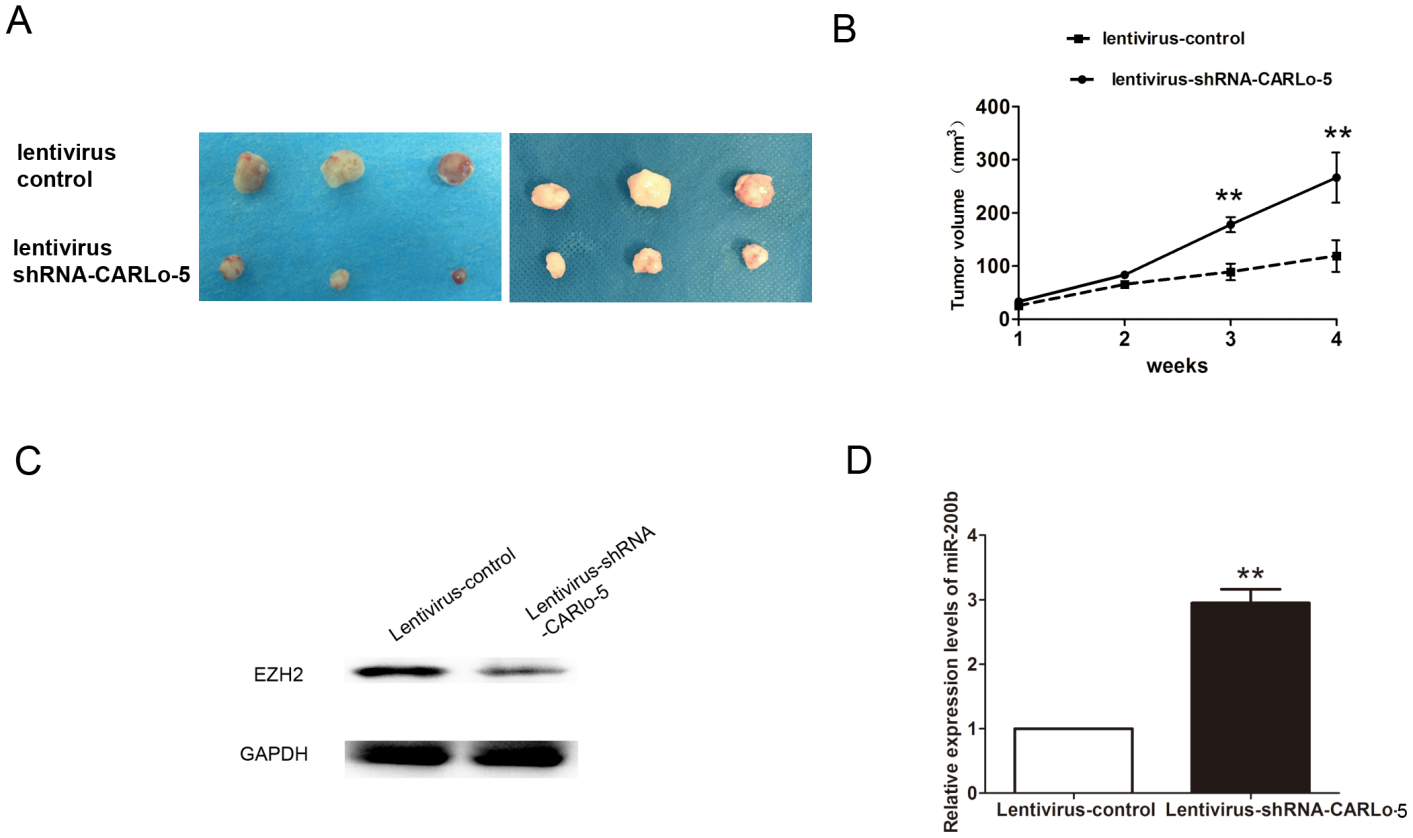
Inhibition of CARLo-5 decreased the tumor growth *in vivo* **(A)** The tumor volume was showed in Lentivirus-shRNA-CARLo-5 group or lentivirus control group after 4 weeks. **(B)** The tumor growth was smaller in Lentivirus-shRNA-CARLo-5 group than that in lentivirus control group. **(C)** The EZH2 protein expression was downregulated in Lentivirus-shRNA-CARLo-5 group than that in lentivirus control group. **(D)** The miR-200b expression was upregulated in Lentivirus-shRNA-CARLo-5 group than that in lentivirus control group, error bars represent the mean ± S.D. of triplicate experiments. **P<0.05.

To further investigated oncogenic activity of CARLo-5 on tumor progression, MHCC97H cells stably expressing lentivirus-shRNA-CARLo-5 or lentivirus control were injected subcutaneously into mice. Tumor volume was measured on a weekly basis and the results suggested that tumor volumes and growth in the lentivirus-shRNA-CARLo-5 group was significantly smaller and slower than that in the lentivirus group (Figure 6A-6B). Moreover, we also demonstrated that EZH2 expression was downregulated, but miR-200b expression was upregulated in the lentivirus-shRNA-CARLo-5 group than that in the lentivirus group (Figure 6C-6D). Thus, these results demonstrated that knockdown of CARLo-5 inhibited tumor growth *in vivo*.

## DISCUSSION

Recently, studies have indicated that dysregulation of lncRNA involved in the biologic processes of tumor [21]. In HCC, Long non-coding RNA CCAT2 functions as an oncogene in hepatocellular carcinoma, regulating cellular proliferation, migration and apoptosis [22]. LncRNA HULC enhances epithelial-mesenchymal transition to promote tumorigenesis and metastasis of hepatocellular carcinoma via the miR-200a-3p/ZEB1 signaling pathway [23]. Liu et al reports that long noncoding RNA FTX inhibited hepatocellular carcinoma proliferation and metastasis by binding MCM2 and miR-374a [24]. A previous study provided evidence that the expression of long non-coding RNA CARLo-5 is upregulated in HCC tissues compared with normal tissues and was associated with disease progression and predicted outcome in hepatocellular carcinoma patients [25]. In the present study, we showed that CARLo-5 was highly expressed in HCC tissues compared with adjacent normal tissues and meanwhile higher CARLo-5 had a poor prognosis in HCC patients. In addition, CARLo-5 silencing inhibited the cell proliferation and cell invasion in MHCC97H and MHC97L cells. *In vivo*, we also found that knockdown of CARLo-5 inhibited tumor growth.

Furthermore, we showed that CARLo-5 played a role in HCC cell EMT process. In our study, CARLo-5 knockdown downregulated ZEB1, Twist1, Vimentin and and upregulated E-cadherin, whereas, the overexpression of CARLo-5 had the reverse effects. Loss of long noncoding RNA FOXF1-AS1 regulates epithelial-mesenchymal transition, stemness and metastasis of non-small cell lung cancer cells via EZH2 [26]. In non-small-cell lung cancer cells, upregulated long non-coding RNA AGAP2-AS1 represses LATS2 and KLF2 expression through interacting with EZH2 and LSD1 [27]. In nasopharyngeal carcinoma, long noncoding RNA H19 regulates EZH2 expression by interacting with miR-630 and promoted cell invasion [28]. In oral squamous cell carcinoma, study showed that long non-coding RNA HOTAIR promotes tumor cell invasion and metastasis by recruiting EZH2 and repressing E-cadherin [29]. In our study, we further confirmed that CARLo-5 was associated with enhancer of zeste homolog 2 (EZH2) by RIP assays.

MiR-200b was a tumor suppressor in HCC, a study reported that MiR-200b/200c/429 subfamily negatively regulates Rho/ROCK signaling pathway to suppress hepatocellular carcinoma metastasis and enhancer of zeste homolog 2 epigenetically silenced tumor suppressor miR-200b to promote liver cancer metastasis [18, 30]. In the study, CARLo-5 was associated with enhancer of zeste homolog 2 (EZH2) and that this association was required for the repression of miR-200b.

In summary, our present study highlighted that lncRNA-CARLo-5 was upregulated in HCC and higher CARLo-5 predicted a poor survival time in patients. We further confirmed that CARLo-5 was associated with enhancer of zeste homolog 2 (EZH2) and that this association was required for the repression of miR-200b. This inhibition of CARLo-5 can be considered as a potential target for the HCC.

## MATERIALS AND METHODS

### Patients and tissue samples

The 87 cases of HCC tissues and adjacent normal tissues samples were obtained from HCC patients underwent radical surgery and collected from March 2010 to April 2014 at Department of Hepatobiliary Surgery, the First Affiliated Hospital of Chinese PLA General Hospital. None of the patients received chemotherapy or radiotherapy before the surgery. The diagnosis and histological grade of each case were confirmed by two professional pathologists independently. The clinicopathological factors such as age, gender, tumor size, American Joint Committee on Cancer (AJCC) stage were analyzed. All samples were immediately frozen in liquid nitrogen and stored at −80°C for further analysis. The study was approved by the Ethics Committee of the First Affiliated Hospital of Chinese PLA General Hospital. Written informed consent was obtained from all patients.

### Cell culture

Three human HCC cells lines (MHCC-97H, SMCC-7721 MHCC97L) and a normal liver cell line (L02) were purchased from Cell Bank of the Chinese Academy of Science (Shanghai, China). The cells were cultured in Dulbecco’s modified Eagle’s medium (Gibco, USA) supplemented with 10 % fetal bovine serum (Gibco, USA), 100 units/ml penicillin, and 100 mg/ml streptomycin. Cultures were incubated at 37 °C in a humidified atmosphere containing 5 % CO2.

### Cell transfection

The siRNAs specifically targeting CARLo-5 were synthesized by Ribobio, Guangzhou, China. The siRNA sequences for CARLo-5 were CARLo-5 siRNA-1 (siCARLo5-1): GGAGGGUGCUUGACAAUAAUU, CARLo-5 siRNA-2 (siCARLo5-2): GAGAAGACCAUAAGAAGAU. The siRNAs specifically targeting EZH2 were synthesized by Ribobio, Guangzhou, China, si-EZH2, 5’-GACUCUGAAUGCAGUUGCUTT-3’. Transfections were performed using the Lipofectamine 2000 kit (Invitrogen, USA) according to the manufacturer’s instructions. The full length CARLo-5 sequence was subcloned into the HindIII and EcoRI sites of pcDNA3.1 vector (Invitrogen, USA) to overexpressed CARLo-5.

### Cell proliferation assay

Cell proliferation assay was carried out using CCK8 cell prolifetion assays kit (Dojindo, Japan) according to the manufacturer’s instruction. MHCC97H and MHCC97L cells transfected with si-CARLo-5 or si-NC and pcDNA3.1-CARLo-5 or pcDNA 3.1 vectors were placed into 96-well plate. After incubation for 24, 48, 72 and 96h, 10 μl CCK-8 assay solution was added to each well. Then, after incubation for another 2 h, optical density (OD) at 450 nm was measured with an enzyme immunoassay analyzer (Thermo Fisher Scientific, Inc., Waltham, MA, USA) to estimate cell proliferation among different groups.

### Cell colony formation assay

Transfected MHCC97H and MHCC97L cells were resuspended and 100 cells/well were seeded into 12-well plates and cultured at 37°C for 2 weeks. Cells were washed with PBS and fixed with 4% paraformaldehyde for 1 h and stained with 0.1% crystal violet for 20 min. The cells colonies were photographed with a digital camera. Experiments were repeated at least three times.

### Cell invasion assays

1 × 10^5^ MHCC97H or MHCC97L cells transfected were seeded into the upper chamber of an insert (8.0 μm, BD, USA). The chamber was precoated with Matrigel (Sigma). The chambers were then incubated for 24 h in culture medium with 10% FBS in the bottom chambers before examination. The cells on the upper surface were scraped and washed away, whereas the invaded cells on the lower surface were fixed by 4% paraformaldehyde for 30 minutes and stain using crystal violet for 10 minutes. Finally, invaded cells were counted by microscope. Experiments were independently repeated in triplicate.

### Quantitative real-time reverse transcription-PCR (RT-PCR)

Total RNA was extracted from cells using Trizol (Invitrogen, USA) according to the manufacturer’s protocol. Reverse transcription was carried out using the PrimeScript^®^ RT Kit (TAKALA, Dalian, China), followed by quantitative PCR in a 7500 Real-Time PCR System (Applied Biosystems, USA) using SYBR^®^ Premix Ex Taq™ II (TAKALA, Dalian, China). The mRNA expression of CARLo-5 or miR-200b was normalized to levels of GAPDH or U6. All results were calculated using the 2^[−ΔΔC(T)]^ method. All experiments were performed in triplicates. The primers for reverse transcription were the following, forward, CARLo-5, forward, 5’-GCCACAAATCAACAACAACAACAACAA-3’, reverse, 5’-AGAGTGATGCCAAGGCTGTTATTGTCAA-3’.GADPH, forward, 5’-GTCAACGGATTTGGTCTGTATT-3’, reverse, 5’-AGTCTTCTGGGTGGCAGTGAT-3’.MiR-200b, forward, 5’-AGCGGCTCATCTAAACAATGG-3’, reverse, 5’-GGCGCACATTCTCTCCGTA-3’.

### Western blotting analysis

Tissues and cell samples were lysed by using RIPA protein extraction reagent (Beyotime Biotechnology, Shanghai, China). Proteins were separated on 10% sodiumdodecylsulfate-polyacrylamide gel electrophoresis (SDS-PAGE), transferred to nitrocellulose membranes (Millipore) and incubated in 4°C for one night with specific antibodies. The membranes were incubated about 1 h using goat anti-rabbit IgG antibody. The bands were visualized by Bio-Rad. Antibodies was used in the study was anti-E-cadherin(1 : 1000 dilutions, Cell Signaling Technology, USA), Vimentin (1 : 1000 dilutions, Cell Signaling Technology, USA), ZEB1(1 : 1000 dilutions, Cell Signaling Technology, USA), and Twist1 (1 : 1000 dilutions, Abcam, USA). Protein levels were normalized to the amount of GAPDH (1 : 3000 dilutions, Cell Signaling Technology, USA) detected on the same blot. Representative data from three independent experiments are shown.

### RNA binding protein immunoprecipitation (RIP) assay

RNA immunoprecipitation was performed using the EZMagna RIP kit (Millipore, Billerica, MA, USA) according to the manufacturer’s protocol. MHCC97H and MHCC97L cells at 80-90% confluency were scraped off, then lysed in complete RIP lysis buffer, after which 100 μl of whole cell extract was incubated with RIP buffer containing magnetic beads conjugated with human anti-EZH2 and anti-H3k27me3 antibody (Cell Signaling Technology, USA), negative control normal mouse IgG (Millipore). Anti-SNRNP70 (Millipore) was used as positive control for the RIP procedure. Samples were incubated with Proteinase K with shaking to digest the protein and then immunoprecipitated RNA was isolated. The purified RNA was subjected to qRT-PCR analysis to demonstrate the LncRNA CARLo-5 expression levels.

### Chromatin immunoprecipitation (ChIP) qPCR analysis

Chromatin Immunoprecipitation (ChIP) Assay Kit (Milipore, Temecula, CA) was used to performed the assays according to manufacturer’s instructions. The antibodies including EZH2 and anti-H3K27me3 (Milipore) were using in the experiment. Control for these antibodies include manufacturer provided IgG and ascitic fluid negative control. Primer design to the miR-200b promoter region was performed based on an identified promoter region (Sigma-Aldrich). The miR-200b was detected by qRT-PCR analysis.

### Xenograft model

The male BALB/c athymic nude mice (n = 3 per group, 3 weeks old) were purchased from Cancer Institute of the Chinese Academy of Medical Science (Beijing, China). 1.5×10^6^ MHCC97H cells stably trasfected lentivirus-shRNA-CARLo-5 or lentivirus control was subcutaneously injected into either side of flank area. After 4 weeks, mice were killed, and tumors volume was evaluated every week. All animal experiments were performed in accordance with the Guide for the Care and Use of Laboratory Animals published by the US National Institutes of Health.

### Statistical analysis

The data analysis was performed using SPSS 17.0 (IBM, USA). Data were presented as mean ± standard deviation, and inter-group differences were assessed for significance using Student’s t test. Survival analysis was estimated using the Kaplan–Meier method and log-rank test. All statistics should be two-tailed, the *p* < 0.05 was considered to indicate a statistically significant difference.
